# Laparoscopic hysterectomy with or without pelvic lymphadenectomy or sampling in a high-risk series of patients with endometrial cancer

**DOI:** 10.1186/1477-7800-3-28

**Published:** 2006-09-13

**Authors:** Susan F Willis, Desmond Barton, Thomas EJ Ind

**Affiliations:** 1 Department of Gynaecological Oncology, Royal Marsden Hospital, Fulham Road, London SW3 6JJ, UK

## Abstract

**Background:**

The purpose of the study was to determine the outcome of all patients with endometrial adenocarcinoma cancer treated by laparoscopic hysterectomy at our institution, many of whom were high-risk for surgery.

**Methods:**

Data was collected by a retrospective search of the case notes and Electronic Patient Records of the thirty eight patients who underwent laparoscopic hysterectomy for endometrial cancer at our institutions.

**Results:**

The median body mass index was 30 (range 19–67). Comorbidities were present in 76% (29 patients); 40% (15 patients) had a single comorbid condition, whilst 18% (7 patients) had two, and a further 18% (7 patients) had more than two. Lymphadenectomy was performed in 45% (17 patients), and lymph node sampling in 21% (8 patients). Median operating time was 210 minutes (range 70–360 minutes). Median estimated blood loss was 200 ml (range 50–1000 ml). There were no intraoperative complications. Post-operative complications were seen in 21% (2 major, 6 minor). Blood transfusion was required in 5% (2 patients). The median stay was 4 post-operative nights (range 1–25 nights). In those patients undergoing lymphadenectomy, the mean number of nodes taken was fifteen (range 8–26 nodes). The pathological staging was FIGO stage I 76% (29 patients), stage II 8% (3 patients), stage III 16% (6 patients). The pathological grade was G1 31% (16 patients), G2 45% (17 patients), G3 24% (8 patients).

**Conclusion:**

Laparoscopic hysterectomy can be safely carried out in patients at high risk for surgery, with no compromise in terms of outcomes, whilst providing all the benefits inherent in minimal access surgery.

## Background

The gold standard for staging and preliminary treatment of endometrial cancer (EC) is surgical [1]. Surgery consists of laparotomy, peritoneal washings, hysterectomy, bilateral salpingo-oophorectomy, and pelvic lymphadenectomy or sampling. The role of lymphadenectomy has been dependant on histology, radiological findings, and local policy.

Traditionally, surgical staging has been carried out through laparotomy. In recent years several studies have demonstrated that a laparoscopic approach to surgery for EC results in outcomes comparable to or better than laparotomy [2-6]. The benefits of laparoscopic surgery for EC may include reduced post-operative pain and analgesic requirements; earlier return of bowel function; shorter hospital stay; and earlier return to daily activities [7-10].

Obese patients and those with medical comorbidities are at greater risk of complications [11]. We describe our experience of laparoscopic surgery for EC in a high risk series of patients referred to a gynaecological cancer centre.

## Methods

A retrospective search of the medical case notes and the Electronic Patient Records provided the data. Besides demographic statistics, patient details recorded included parity, body mass index (BMI) and any comorbid conditions. The type of operation, intra- and post-operative complications and details of histopathological grade and stage were also recorded.

All patients had pre-operative pelvic and abdominal CT or MRI scan, and each case was discussed at a regular Multi-Disciplinary Team Meeting. Operations were performed by a gynaecological oncologist or a sub-specialty trainee with the consultant in attendance. Histological reporting included depth of myometrial invasion and nodal status.

A standard 4-port approach was employed. All patients had peritoneal washing taken at the start of the procedure. The policy is to perform laparoscopically assisted vaginal hysterectomies. However, due to obesity and poor access in this group of patients, this was not always possible. In some cases the cervix was only visible with a hysteroscope; in these women a laparoscopic total hysterectomy was performed using a MacCartney tube. If there is macroscopic intraperitoneal disease, then our policy is to convert to laparotomy.

Pelvic lymphadenectomy was performed in those who were randomised to the ASTEC trial [12]. For those patients who declined to enter the ASTEC trial, then all those with stage Ic grade 3 cancers and above on radiological and histopathological review underwent lymphadenectomy. Lymph node sampling was performed instead of pelvic lymphadenectomy in cases with severe co-morbidity, or not at all. By sampling we mean a limited lymphadenectomy, without stripping the vessels. Para-aortic lymphadenectomy for EC was not routinely performed in this series.

## Results

The median age was 62 years (range 38–82 years). The average BMI was 31 (range 19–67). Nine patients (24%) were nulliparous.

This group of patients had a number of comorbid conditions; fifteen patients (40%) had a single comorbid condition, seven (18%) had two, and a further seven patients (18%) had more than two.

Twenty four patients (63%) were overweight with a BMI of 25 or more (table 1). Seventeen patients (45%) had a BMI greater than 30. Hypertension was present in fifteen patients (40%); eight patients (21%) had previous intraabdominal pathology or surgery; and five patients (13%) had cardiovascular disease (table 1). Other comorbidities included thyroid disease, breast cancer, thromboembolic disease, respiratory disease and diabetes mellitus (table 1).

**Table 1:** Comorbid conditions

Comorbidity	Patients
Overweight (BMI >25)	24 (63%)
Obese (BMI >30)	17 (45%)
Hypertension	15 (40%)
Severe endometriosis/previous peritonitis/previous abdominal surgery	8 (21%)
Cardiovascular disease	5 (13%)
Thyroid disease	4 (11%)
Breast cancer	5 (13%)
Thromboembolic disease	3 (8%)
Respiratory disease	3 (8%)
Diabetes mellitus	2 (5%)

There were 27 (71%) laparoscopic-assisted vaginal hysterectomies and 11 (29%) total laparoscopic hysterectomies. Lymphadenectomy or lymph node sampling was performed in 25 patients (66%). The median node count for lymphadenectomy was 15 (range 8–26 nodes). Positive nodes were found in four patients (11%), two who had lymph node sampling and two who had lymphadenectomy.

The surgical stage of the patients is shown in table 2. Twelve patients (31%) had grade 1 disease, 17 (45%) had grade 2 disease, and the remaining nine (24%) had grade three lesions. The pathological type was predominantly endometrioid (table 3). Three patients (8%) had positive peritoneal washings. Myometrial invasion and cervical involvement are shown in table 4.

**Table 2:** FIGO stage on final histology

Stage		Sub-stage	
I	29 (76%)	Ia	11 (29%)
		Ib	14 (37%)
		Ic	4 (11%)
II	3 (8%)	IIa	1 (2%)
		IIb	2 (5%)
III	6 (16%)	IIIa	2 (5%)
		IIIb	0
		IIIc	4 (11%)
IV	0		

**Table 3:** Pathological type

Pathological type	Patients
Endometrioid	30 (79%)
Serous papillary	3 (8%)
Clear cell/mixed mullerian/mixed endometrioid +serous papillary/mixed serous papillary + clear cell/intraepithelial	5 (13%)

**Table 4:** Myometrial and cervical involvement

Myometrial invasion		Patients
	None	9 (24%)
	<50%	16 (42%)
	>50%	13 (34%)
Cervical involvement		
	Stromal	2 (5%)
	Glandular	1 (2%)

The median operative duration was 210 minutes (range 70–360 minutes). The median estimated blood loss was 200 ml (range 50–1000 ml). Blood transfusion was necessary in two patients (5%). None of the laparoscopic cases needed to be converted into open procedures and there were no incidents of intra-operative visceral damage. The median length of stay was four post-operative nights (range 1 – 25 nights).

There were a total of eight post-operative complications, two of which were major (table 5).

**Table 5:** Post-operative complications

Major complications		Patients
	Renal failure secondary to diclofenac use (reversible)	1
	Infected lymphocyst	1
Minor complications		
	Unexplained vomiting	1
	Superficial umbilical wound breakdown	1
	Extensive bruising of anterior abdominal wall	1
	Pyrexia of unknown origin	3

## Discussion

This series of patients had a large number of comorbidities, particularly with regard to obesity. It might be expected that these patients would have more intra-operative complications (due to difficulties with access) and more post-operative complications (due to comorbid conditions) than patients with fewer risk factors for surgery. Here we have demonstrated that this is not the case. These results show that EC patients perceived to be poor risk for a major surgical procedure can be safely staged using laparoscopic methods. It has been shown that obese patients with EC can benefit from surgery via the laparoscopic route compared with laparotomy in terms of a reduced blood loss, less post-operative pain and a shorter hospital stay [13]. A recent small audit of 4 morbidly obese (BMI >40) women found that laparoscopic total hysterectomy was safe in these patients [14].

With regard to other published series of EC patients operated on laparoscopically, our data compare favourably in terms of post-operative complications, intra-operative blood loss, duration of surgery, and length of post-operative stay [7,13,15]. The two major post-operative complications seen here (renal failure secondary to diclofenac use and an infected lymphocyst) were both successfully managed conservatively. The safety and feasibility of laparoscopic hysterectomy in this group of high-risk patients is demonstrated by the zero conversion to laparotomy, and the absence of intraoperative complications. Postoperative blood transfusion was minimal, and where blood loss was greater than 300 ml, this invariably occurred in the vaginal part of the hysterectomy.

Lymph node counts following pelvic lymphadenectomy were similar to those seen in other studies utilising the laparoscopic approach [7,13,15]. Lymph node sampling was performed in patients in whom a shorter anaesthetic was desirable. With increasing experience of laparoscopic techniques, operating times will be further reduced. In any case, all patients with positive lymph nodes following either sampling or pelvic lymphadenectomy are offered adjuvant, extended field radiotherapy. The role of lymphadenectomy in women with endometrial cancer will become clearer when the results of the ASTEC study [12] are reported.

We believe our study is important, as obesity is a growing epidemic in the UK and other developed nations. It has been shown that a cohort of obese patients with EC have a greater risk of hypertension, diabetes and pulmonary disease than patients with EC who are not obese [16]. As we see more and more obese patients in our clinics, the challenge to gynaecologists and anaesthetists in treating these patients with multiple medical co-morbidities is increasing.

These data are limited by patient numbers. Now that a laparoscopic approach is established in the staging and primary treatment of EC, we can continue to build on our early experience.

## Conclusion

Women with EC are often high-risk surgical candidates. Laparoscopic hysterectomy can be safely carried out in these patients with no compromise in terms of outcomes, whilst providing all the benefits inherent in minimal access surgery.

## Competing interests

The author(s) declare that they have no competing interests.

## Authors' contributions

TEJI and DB performed the procedures. SFW collated the information and drafted the manuscript. All authors read and approved the final manuscript.
